# DNATCO v5.0: integrated web platform for 3D nucleic acid structure analysis

**DOI:** 10.1093/nar/gkaf1491

**Published:** 2026-01-06

**Authors:** Jiří Černý, Michal Malý, Paulína Božíková, Terezie Prchalová, Jakub Svoboda, Lada Biedermannová, Bohdan Schneider

**Affiliations:** Institute of Biotechnology of the Czech Academy of Sciences, Průmyslová 595, Vestec 25250, Czech Republic; Institute of Biotechnology of the Czech Academy of Sciences, Průmyslová 595, Vestec 25250, Czech Republic; Institute of Biotechnology of the Czech Academy of Sciences, Průmyslová 595, Vestec 25250, Czech Republic; Institute of Biotechnology of the Czech Academy of Sciences, Průmyslová 595, Vestec 25250, Czech Republic; Institute of Biotechnology of the Czech Academy of Sciences, Průmyslová 595, Vestec 25250, Czech Republic; Institute of Biotechnology of the Czech Academy of Sciences, Průmyslová 595, Vestec 25250, Czech Republic; Institute of Biotechnology of the Czech Academy of Sciences, Průmyslová 595, Vestec 25250, Czech Republic

## Abstract

As the number and complexity of RNA and DNA structures continue to expand, there is a growing need for robust yet accessible tools that support their accurate interpretation, validation, and refinement. We present DNATCO v5.0 (dnatco.datmos.org), an interactive web application for comprehensive structural analysis of nucleic acids. DNATCO integrates the NtC dinucleotide conformational classes and the CANA structural alphabet to provide an intuitive, geometrically complete description of local backbone and base orientations, complemented by interactive visualization of base pairing. The platform performs quantitative validation of conformational similarity and covalent bond lengths and angles, using newly established nucleic-acid valence-geometry standards. Quantitative validation encompasses the confal score and scattergrams mapping the fit between experimental electron density and geometry similarity to the closest NtC class. All outputs are downloadable. Integrated diagnostic tools help users identify unusual or problematic regions, explore alternative conformations, and generate torsion-restraint files for downstream. DNATCO v5.0 is implemented entirely client-side via WebAssembly, ensuring fast performance and preserving data privacy, and supports both PDB and user-provided structural models. By combining a rigorous geometric framework with an approachable interface, DNATCO enables both non-experts and specialists to evaluate nucleic-acid structures with greater confidence and to improve models in ways that support accurate biological interpretation.

## Graphical Abstract

**Graphical Abstract:**
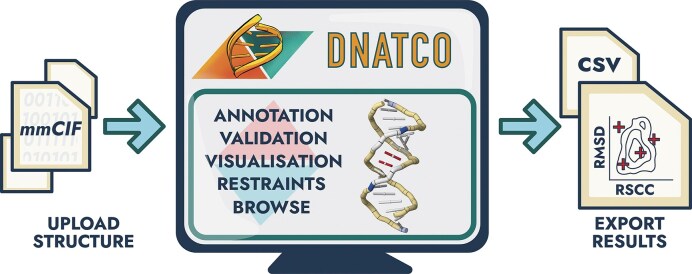

## Introduction

Nucleic acids (NAs) are fundamental to life, serving as the blueprints for genetic information storage and its translation into functional molecules. These vital roles are intrinsically linked to their three-dimensional (3D) structures. Indeed, breakthroughs in molecular biology have often paralleled our understanding of NA structural features. Landmark discoveries, such as the double-helical structure of DNA by Watson, Crick, Franklin, Gosling, and Wilkins in 1953 [1–3], and the determination of ribosomal particle 3D structures [4–7], underscore the impact of structural insights.

The accessibility of experimental NA and protein structures has been a cornerstone for structural biologists since the 1970s, beginning with the establishment of the Protein Data Bank (PDB) [8]. This was further enriched by the specialized Nucleic Acid Database (NDB) [9]. These foundational resources paved the way for the emergence of numerous specialized databases and web services. Tools like MC-Annotate [10], Curves+ [11], NUPACK [12], ClaRNA [13], wDSSR [14], DNATCO [15], DNAproDB [16], watNA [17], ONQUADRO [18], SimRNAweb [19], RNAtango [20], and the recently introduced Nucleic Acid Knowledge Base [21] all highlight the community’s ongoing need for sophisticated NA analysis such as that initiated by automated refinement via PDB-REDO [22].

**Figure 1.**
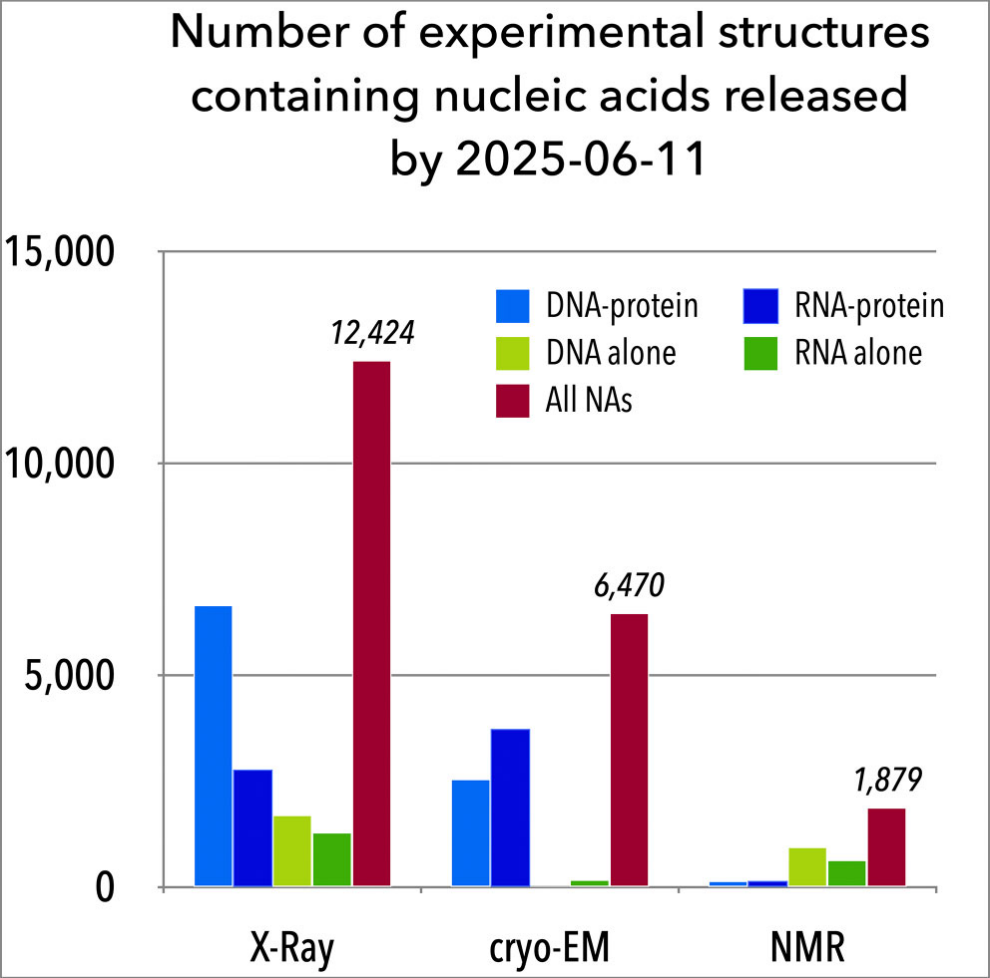
Histogram of number of nucleic acid structures in the Protein Data Bank [23] (data as of 11 June 2025), classified by determination method: X-ray crystallography, cryo-EM, and solution NMR, with no quality or resolution filters applied.

Advances in experimental and computational methodologies have dramatically expanded our understanding of nucleic acid structures. As shown in Fig. 1, the Protein Data Bank [23] currently archives structures of NAs predominantly solved by X-ray crystallography, with a substantial proportion derived from solution nuclear magnetic resonance (NMR) and cryo-electron microscopy (cryo-EM). Since the 1950s, X-ray crystallography has been paramount, providing most atomic-resolution details for NA structures [24]. Complementary techniques such as neutron diffraction [25] and NMR spectroscopy [26] have also yielded invaluable insights. More recently, cryo-EM [27, 28] has seen dramatic improvements, now achieving resolutions comparable to X-ray crystallography for large macromolecular complexes [29, 30]. Of various computational approaches, molecular dynamics simulations provide the most information on the time development of NA structures [31]. Despite that challenges persist regarding the volume and quality of data required to train complex models for predicting RNA 3D structures [32], deep learning and artificial intelligence show promise for future advancements in NA structure determination [33].

The proliferation of structural data has necessitated the parallel development of robust annotation and validation tools. These tools often rely on precise parameter limits derived from experimentally determined structures. Early efforts established base valence parameters from high-resolution crystal structures [34, 35] from the Cambridge Structural Database [36], later independently confirmed for the bases [37] and sugar-phosphate valence parameters [38]. A fundamental aspect of NA 2D and 3D structures, base pairing, was systematically cataloged by Saenger [39], with a more systematic classification, the Leontis–Westhof schema [40, 41], becoming the most widely adopted approach.

Furthermore, conformational characteristics, initially explored through torsional space analysis of the first NA structures [42], have seen extensive re-evaluation with orders of magnitude more data available [43–47]. Most methods for classifying NA conformational space in torsional dimensions have focused on dinucleotides or similarly sized fragments. A prominent example is the concept of dinucleotide conformational classes (NtC), defined by specific ranges of 12 torsion parameters [47]. Currently, 96 recognized NtC classes are organized into 14 codes within the Conformational Alphabet of Nucleic Acids (CANA). To quantitatively assess the fit between analyzed fragments and these NtC-defined standards, we developed two key measures: confal (for conformational validation) and RMSD. The confal score quantifies similarity in the 12-parameter space between an analyzed step and its assigned NtC class reference, with values ranging from 0 (no match) to 100 (perfect match). Concurrently, RMSD represents the root mean square deviation in Cartesian space between the analyzed step and the reference. This RMSD measure of structural similarity is further utilized in 2D RSCC/RMSD plots, which visually depict how well different parts of a model structure align with known NtC geometries and experimental electron density maps. These concepts are central to the DATMOS web service, available at dnatco.datmos.org, which provides detailed reports on the structural features of individual 3D structures containing NAs, keeping pace with the rapid influx of new experimental and computational data.

The newly reported DNATCO version 5.0 represents a substantial advancement over the previously published version 3.2 [48], significantly enhancing its data flow, information content, and layout: (i) Browser-based WebAssembly (WASM) Architecture: all structure analyses are now executed directly on the user’s computer, eliminating the need to upload sensitive user data to remote servers. This ensures enhanced privacy and efficiency. (ii) Expanded analysis capabilities: in addition to the established analysis of nucleic acid conformations into NtC classes [47], DNATCO v5.0 now integrates results from base pair identification using the widely recognized Leontis–Westhof nomenclature [40, 41]. As a part of the DNATCO Mol* graphical panel, we also introduce, to the best of our knowledge, a unique graphical representation of base pairs, providing an intuitive insight into structurally the most important molecular regions. We also provide valence geometry validation. (iii) Interactive validation tools: we provide powerful, interactive RSCC/RMSD graphs, offering deeper insights into structural quality. (iv) Improved user interface and organization: the structural analysis features have been rearranged into several intuitive logical blocks: Annotation, Validation, Refinement, Browse, and context-sensitive Help. (v) Enhanced data export: analysis results are now downloadable in multiple formats, including an mmCIF format [49] extended with NA-specific categories, facilitating seamless integration with other bioinformatics workflows.

## Materials and methods

### DNATCO web application

**Figure 2.**
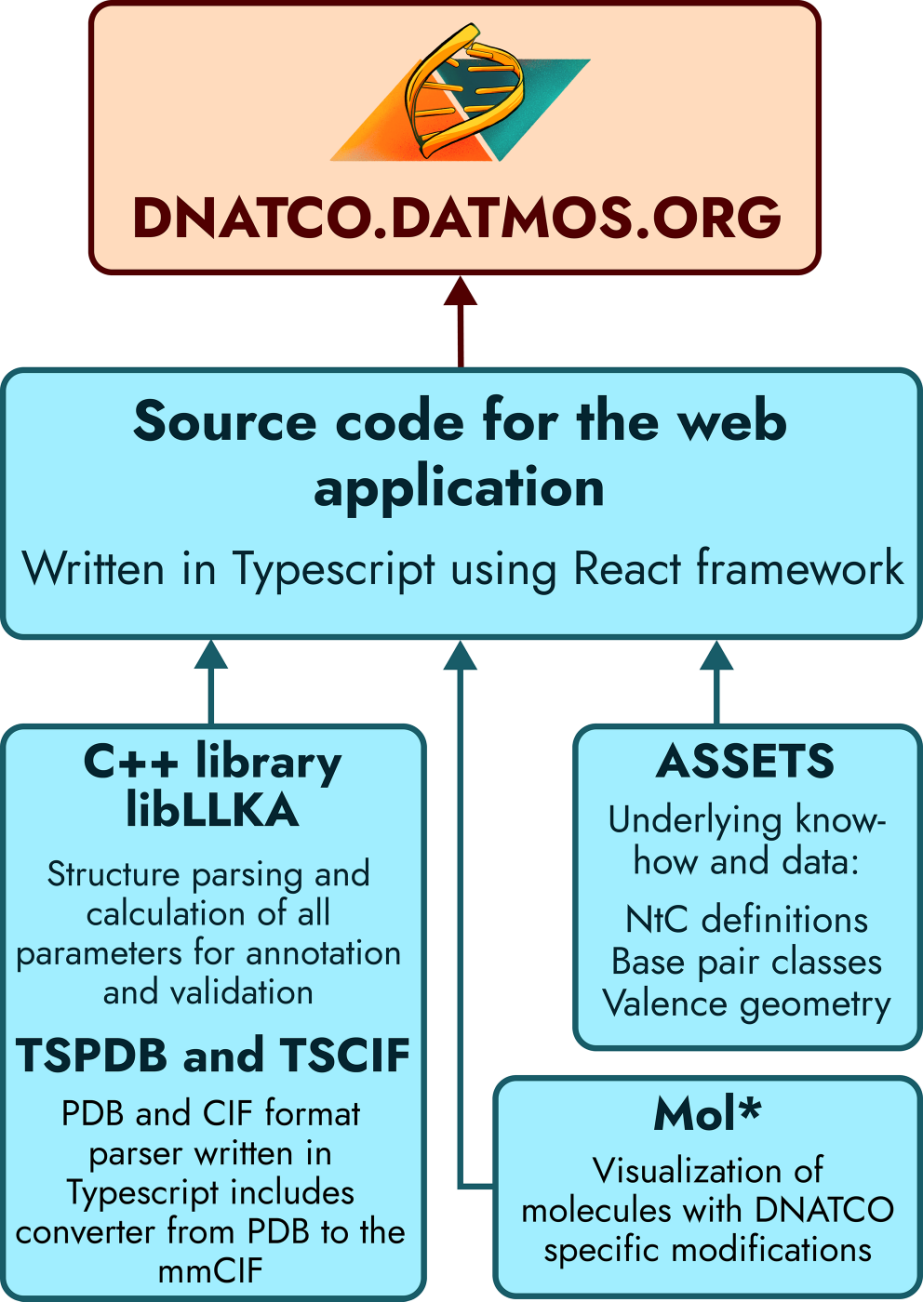
Schematic overview of the DNATCO application architecture. A detailed version is provided in the Supplementary Fig. S1.

The DNATCO v5.0 web application (https://dnatco.datmos.org) seamlessly combines high-performance processing of nucleic-acid structural data, interactive molecular visualization, and a comprehensive knowledge base within a TypeScript-driven React interface (Fig. 2). The server hosts both the client application and its precomputed datasets while exposing additional backend services for validation and optional analyses.

At its core, the client-side interface is implemented in TypeScript using React, which enables modular, reusable UI components and enhances maintainability. Styling is handled by Tailwind CSS, offering a utility-first approach for rapid, custom design.

The structural-processing engine is written in C++ with accompanying C bindings to facilitate integration with other languages. Both the core library and example CLI/GUI programs for batch analysis are available under the MIT license at https://github.com/cernylab/libllka. This module relies on Eigen3 for linear algebra and fastfloat for parsing numerical data. We compile it to WebAssembly via Emscripten, allowing computationally intensive tasks to execute directly in the browser—improving performance, reducing latency, and preserving user data privacy.

Visualization is powered by a customized Mol* Viewer [50], augmented to display DNATCO-specific features such as confal pyramids—color-coded by backbone conformation class and sized by quality score—and the novel NtC-tube representation. The NtC tube traces the backbone via C5′ and O3′ atoms, yielding a smooth, twist-resistant ribbon that highlights chain irregularities and, for large assemblies, focuses attention on backbone geometry by omitting bases.

On the server side, an Apache 2.4 instance runs within an OpenStack-managed cloud (ELIXIR CZ infrastructure) on CentOS Stream, ensuring stability and high availability over HTTPS. The backend maintains precomputed annotations and validation results for all PDB entries, updating weekly as new structures are released. Optional real-space correlation coefficient (RSCC) calculations use the phenix.real_space_correlation module of Phenix v1.18.2 [51], and non-standard file corrections leverage the MAXIT suite ([52] MAXIT version 10.000. https://sw-tools.rcsb.org/apps/MAXIT). To protect user privacy, custom files are only transmitted for these optional services when explicitly requested.

### Nucleic acids reference sets

To establish rigorous empirical standards for nucleic-acid valence geometry, we have constructed a strict high-resolution PDB-NA Reference Set [53]. All PDB X-ray entries with crystallographic resolution ≤1.8 Å that contain DNA or RNA were clustered by nucleotide-sequence similarity. From each cluster, the highest-quality chain was then selected using an extended composite quality score (CQS) integrating resolution, R_free_, clashscore, RSCC, RSR, and model completeness. Only chains with CQS <15 were retained for this stringent set. Individual residues were then filtered by NtC-based conformational quality and density criteria (Confal ≥60, RSCC ≥0.8, RMSD ≤0.5 Å), and MolProbity-based quality filters were applied to remove residues affected by clashes or other local model inconsistencies. This combined filtering produced 3202 DNA residues and 2544 RNA residues, each belonging to dinucleotide steps or longer contiguous segments with well-defined covalent and conformational geometry. The resulting PDB-NA Reference Set provides a robust empirical foundation for natural nucleic-acid stereochemical variability and forms the basis for the probability percentile score (ProSco) and the Preferred–Allowed–Of Concern classification used in our valence-geometry validation protocol.

In parallel with the strict PDB-NA Reference Set, we constructed broader general-purpose high-quality reference sets designed for modeling, benchmarking, and machine-learning applications. All nucleic-acid–containing X-ray entries with resolution up to 3.5 Å and available reflection data were collected, clustered by sequence similarity, and assessed using the same extended CQS scoring. For each sequence cluster, we selected up to two highest-quality representative chains—one from an unbound nucleic acid when available, and one from a nucleic acid–protein complex when available—so that the reference sets capture geometry characteristic of both structural contexts. These chains are forming the first part of this general-purpose dataset.

To capture realistic interaction environments, we generated a second complementary set that includes all nucleic acid residues within 4 Å of each reference chain, with the reference residues and their contacting partners listed together. The underlying rationale is that structural fragments immediately surrounding high-quality nucleic-acid chains are themselves typically well resolved and therefore valuable for learning or benchmarking local interaction contexts. Together, these two sets provide high-fidelity structural examples suitable for benchmarking modeling and refinement protocols at typical crystallographic resolutions, as well as for training machine-learning models that require accurate residue-level geometry and realistic local environments.

All datasets produced in this work are available for download from the DNATCO website (https://dnatco.datmos.org/app/reference-sets). These include the high-quality chain-level reference sets derived from sequence-non-redundant clusters at resolutions up to 3.5 Å, the corresponding residue-level lists together with all nucleic-acid residues within 4 Å of each reference chain to capture realistic interaction environments, and the strict high-resolution PDB-NA Reference Set containing residue-level data filtered by NtC conformational quality and electron-density support. Together, these datasets provide comprehensive standards for nucleic-acid validation, benchmarking, refinement, and machine-learning training.

### Valence geometry validation

DNATCO v5.0 implements the nucleic-acid valence-geometry validation protocol developed by the Nucleic Acid Valence Geometry Working Group and fully described in [53]. Each bond length and bond angle in a validated nucleic-acid structure is evaluated against the empirical distributions obtained from the high-resolution PDB-NA Reference Set (see the “Nucleic acid reference sets” section) and from the CSD small-molecule database.

For every geometry parameter, the empirical distribution from the PDB-NA Reference Set is converted to a ProSco. Values with ProSco ≥5 define the central region containing 95% of observations in the reference set. In parallel, mean ± 3σ intervals from the CSD data are computed. The Preferred range for each parameter is defined by combining both sources, using the more permissive of the two boundaries at each side to account for differences between macromolecular and small-molecule environments.

Values outside the Preferred interval but not exceeding a non-parametric standard-score (Z’) threshold of ±5 are classified as Allowed. Parameters more extreme than this threshold are flagged as Of Concern. The ±5 limit was determined empirically from manual inspection of all high-resolution distributions and reflects the observed variability in biological nucleic acids rather than a classical statistical *z*-score interpretation.

### Base pair classification

**Figure 3.**
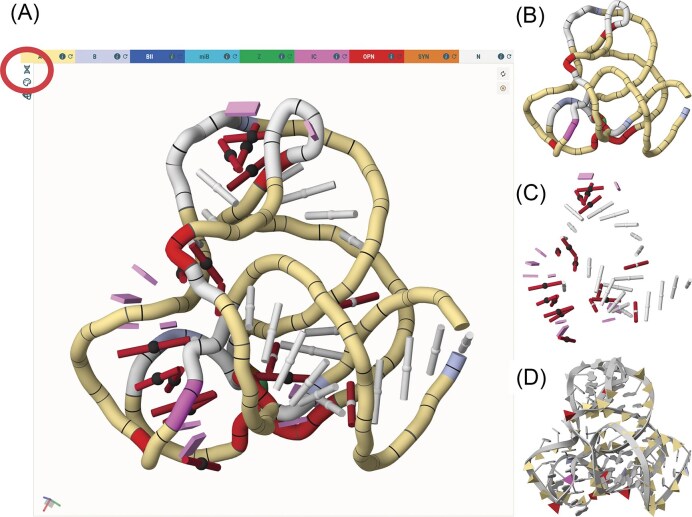
Visualization modes in DNATCO v5.0. The example structure is an 80-nt fragment of 23S rRNA (PDB code 4qvi [55]). (**A**) Default view displaying both the NtC tube and a base-pair ladder, with the view-switch icons (highlighted by the red circle). (**B**) NtC tube only. (**C**) Base-pair ladder only. (**D**) Cartoon representation highlighting NtC classes as colored pyramids.

In DNATCO v5.0, base‐pair annotations are generated using the FR3D program [54] (version 0.0.2, 24 June 2025; commit f5c3bc3 on the “latest” branch of https://github.com/BGSU-RNA/fr3d-python). We adopt the Leontis–Westhof nomenclature to describe base‐pair topologies, combining the three interacting edges—Watson–Crick (W), Hoogsteen (H), and sugar (S)—with the base identities (G, A, C, U/T) and the *cis/trans* orientation of the glycosidic bonds. For example, the canonical G–C Watson–Crick pair is designated cWW_G-C, whereas a frequently observed noncanonical interaction between the Watson–Crick edge of U and the Hoogsteen edge of A in a *trans* orientation is labeled tWH_U-A. Note that tWH_A-U is a distinct pair that occurs only rarely. A new graphical feature in DNATCO v5.0 visually distinguishes base-pair classes: canonical pairs (G–C and A–U/T) appear as white rods, while noncanonical pairs are shown as dark red rods. An alternative, detailed scheme colors the rods by the interacting edges of noncanonical pairs—yellow for Watson–Crick, blue for Hoogsteen, and red for sugar. *Trans*-oriented pairs are marked by a small black sphere between the bases, and unpaired residues by pink tiles (Fig. 3).

### Manuscript preparation

We used ChatGPT (OpenAI, 2024; https://chat.openai.com/chat) to improve the fluency and clarity of the initial draft, particularly in polishing language written by non-native speakers. The authors then conducted a thorough review and revision to ensure the final text is accurate, coherent, and fully reflects our scientific intent.

## Results and discussion

### Overview of the DNATCO web application

DNATCO v5.0 offers a redesigned web interface that makes comprehensive nucleic acid structural knowledge accessible to both novice learners and experienced researchers. The updated C++ structure–processing module accelerates annotation and validation by over 50 times compared to the previous version. A unified code supports sustainable development and enables integration of DNATCO functionality into external tools and services. Figure 2 presents a simplified application schematic; a detailed diagram is available in the Supplementary Material. It performs all computations based on our C++ library compiled to WebAssembly (WASM). By offloading the core functionality to the user’s browser, DNATCO ensures the confidentiality of user-provided data and drastically reduces latency, making analysis not only more secure but also significantly faster. This change leverages the power of client-side technologies to deliver high-performance processing directly in users' environments, minimizing the need for server infrastructure.

DNATCO analyzes public or user-supplied structures in mmCIF or PDB format [56]. All core calculations run locally, ensuring that input files remain on the user’s computer. If a structure contains non-standard features that cannot be processed locally, the user is prompted to upload it to the DNATCO server. In that case, DNATCO invokes the MAXIT suite ([52], MAXIT version 10.000. https://sw-tools.rcsb.org/apps/MAXIT) to correct format inconsistencies; these server-side corrections are required because MAXIT cannot be distributed with DNATCO v5.0.

DNATCO v5.0 organizes structural analyses into six intuitive sections—Home, Annotation, Validation, Refinement, Downloads, and Browse—as described below. Each section provides navigation links in the upper-left corner, including context-specific Help and Downloads tabs. Footer links provide additional resources—How to Cite, Contact, and Help, as well as access to Version History (to explore previous DNATCO releases) and Resources (scripts and nucleotide conformer tables). This design ensures that both novice and expert users can efficiently validate and refine NA structures using DNATCO.

### Case studies

To illustrate how DNATCO supports structural interpretation, validation, and refinement of nucleic-acid models, we discuss two representative examples: the metabolite-bound PreQ1 riboswitch (PDB ID 6vui; [57]) and the nucleosome core particle DNA (PDB ID 6jxd; [58]). Both highlight distinct challenges—RNA tertiary-motif diversity and DNA deformation by protein binding—and show how NtC annotations, valence-geometry analysis, and refinement tools converge to reveal and correct structural issues. A fully guided walkthrough using an 80-nt 23S rRNA fragment (PDB 4qvi [55]) is provided in the Supplementary Material in which Supplementary Fig. S2 depicts the DNATCO Home Page, Supplementary Fig. S3 shows the Conformation tab on the Annotation page, Supplementary Fig. S4 shows the Overall Quality tab on the Validation page, Supplementary Fig. S5 is an illustration of the Similarity plot, Supplementary Fig. S6 displays RSCC/RMSD plot tab, Supplementary Fig. S7 shows Bond Lengths & Angles tab, and Supplementary Fig. S8 shows an intuitive explanation of the Connectivity plot.

#### Riboswitch (6vui): identifying conformational motifs and irregularities

**Figure 4.**
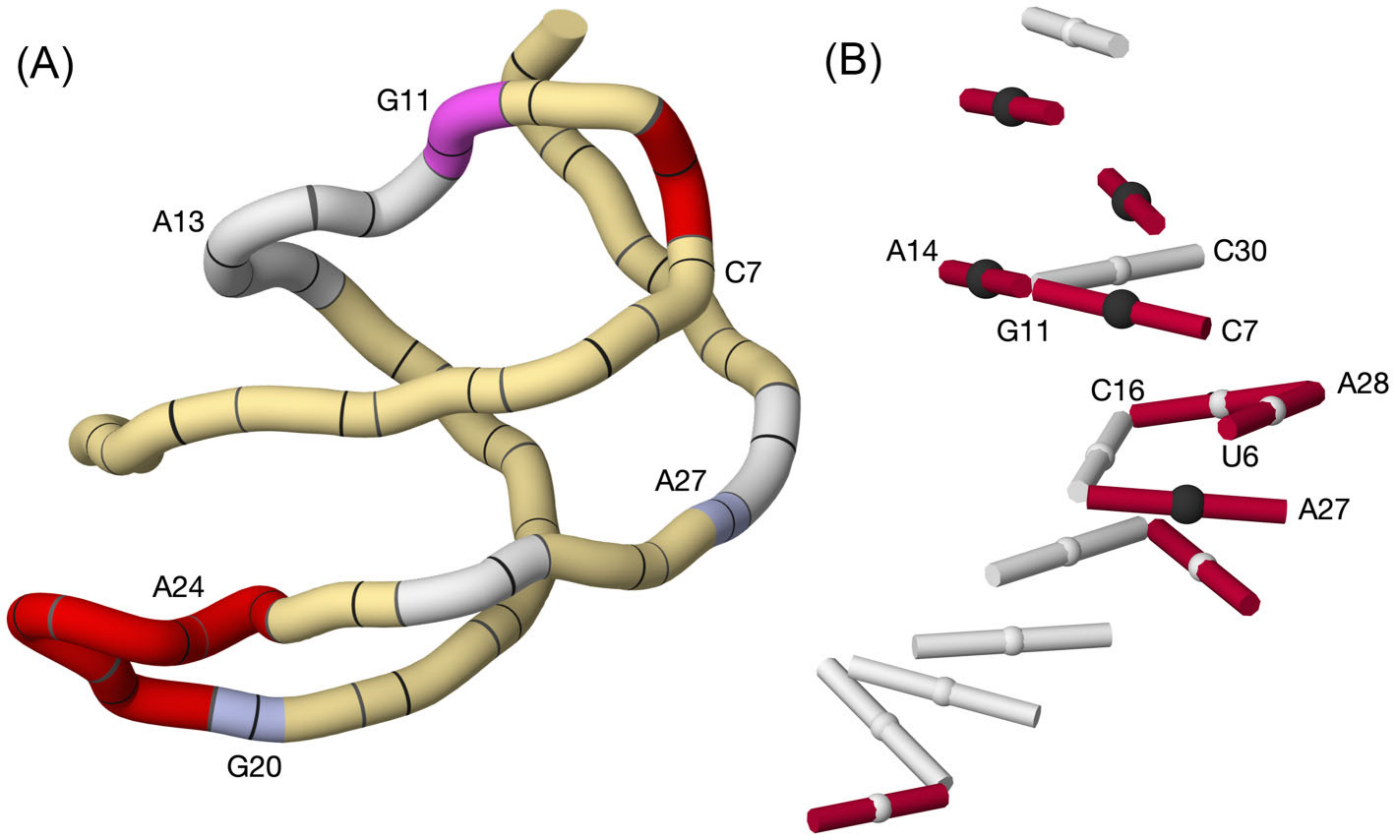
Molecular graphics of the PreQ1 riboswitch (PDB 6vui; [57]). (**A**) NtC-tube representation, color-mapped by dinucleotide conformation: A-form segments in light yellow, loop C7–G11 in magenta, and G20–A24 in red to indicate conformational hotspots. (**B**) Base-pair ladder view with canonical *cis*-Watson–Crick/*cis*-Watson–Crick (cWW) pairs in white, non-canonical pairs in dark red; *trans*-oriented pairs are marked by black spheres between bases, and unpaired bases by pink tiles.

Loading the riboswitch model immediately displays its NtC-colored backbone, with the dominant A-form helical scaffold evident from the long yellow stretches corresponding to AA00 and AA08 classes. This global helical character is punctuated by conformational hotspots readily visible in the tube representation: magenta segments marking the C7–G11 loop and red segments at G20–A24, each signaling dinucleotides with unstacked or highly displaced bases (Fig. 4A).

Interactive selection of these segments highlights the corresponding dinucleotide in atomic detail and overlays the closest NtC conformer, allowing direct comparison between observed and canonical torsion patterns. This quickly reveals why several residues in the C7–G11 region remain unassigned (NtC = NANT): their torsions occupy regions remote from known NtC clusters (RMSD >0.8 Å).

Using the base-pair visualization tools, the riboswitch’s intricate tertiary network becomes apparent. Most pairs follow canonical cWW geometry, yet several non-canonical and trans-oriented interactions appear, including the tWH_C-G pair between C7 and G11 and the multi-edge interactions formed by G11 with both A14 and C30. A staircase of alternating canonical and non-canonical pairs links A27–G5–C16–A28–U6, an arrangement revealed unambiguously by the base-pair ladder view (Fig. 4B).

**Figure 5.**
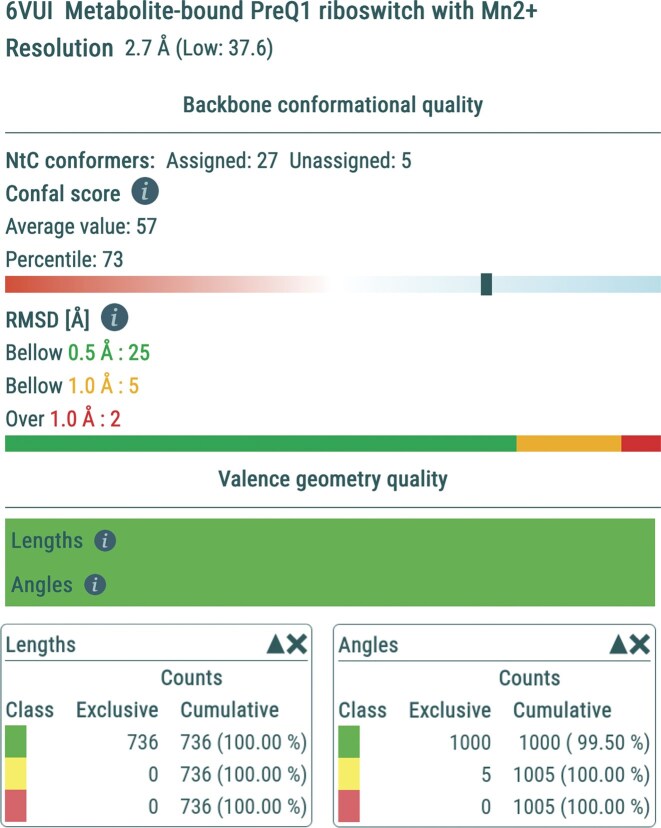
Quality assessment of the PreQ1 riboswitch structure (PDB 6vui; [57]) as shown by the Overall Quality tab of the Validation page. The confal conformational score places the model in the 73rd percentile. Only two dinucleotide steps deviate by >1.0 Å RMSD from their assigned NtC geometries. Valence bond lengths and angles lie within expected ranges with no outliers.

Quality assessment confirms the high accuracy of the deposited model: almost all torsions, NtC assignments, and valence-geometry parameters lie within typical ranges (Fig. 5), and RSCC values are uniformly high. Only a small cluster of residues in the G11–A13 region form genuine outliers, consistent with the structural complexity of this motif.

#### Nucleosome DNA (6jxd): identifying overrepresented conformers and refinement targets

In contrast to the riboswitch, the nucleosome DNA adopts a predominantly B-form geometry, as shown by the extensive blue coloring and frequent BB00, BB04, and BB07 assignments. However, DNATCO immediately highlights a striking feature: an unusually large fraction of BII steps (BB07), appearing 42 times—roughly threefold the expected ∼10% frequency for nucleosomal DNA [59].

Inspection shows several stretches of consecutive BII geometries, including an extremely rare BII stretch of four nucleotides in chain I (G–7 to C–4) and chain J (A–38 to A–35). DNATCO’s stepwise overlay reveals that many of these BII assignments are plausible, yet others sit near the boundary of known conformational space, suggesting potential overfitting or density-driven ambiguity.

Unassigned steps (NtC = NANT) appear predominantly in regions where torsions deviate from known NtC clusters despite close Cartesian similarity. For example, the dinucleotide C(–58)–T(–57) aligns within 0.5 Å of BB10 yet differs by >70° in β and γ torsions. These cases are ideal candidates for manual correction or targeted refinement, supported by DNATCO’s torsion-restraint generation.

Valence-geometry analysis shows that nearly all bond lengths and angles fall within Preferred or Allowed intervals. The interactive probability-distribution plots reveal no systematic distortions, supporting the idea that the model is geometrically consistent but torsionally perturbed.

**Figure 6.**
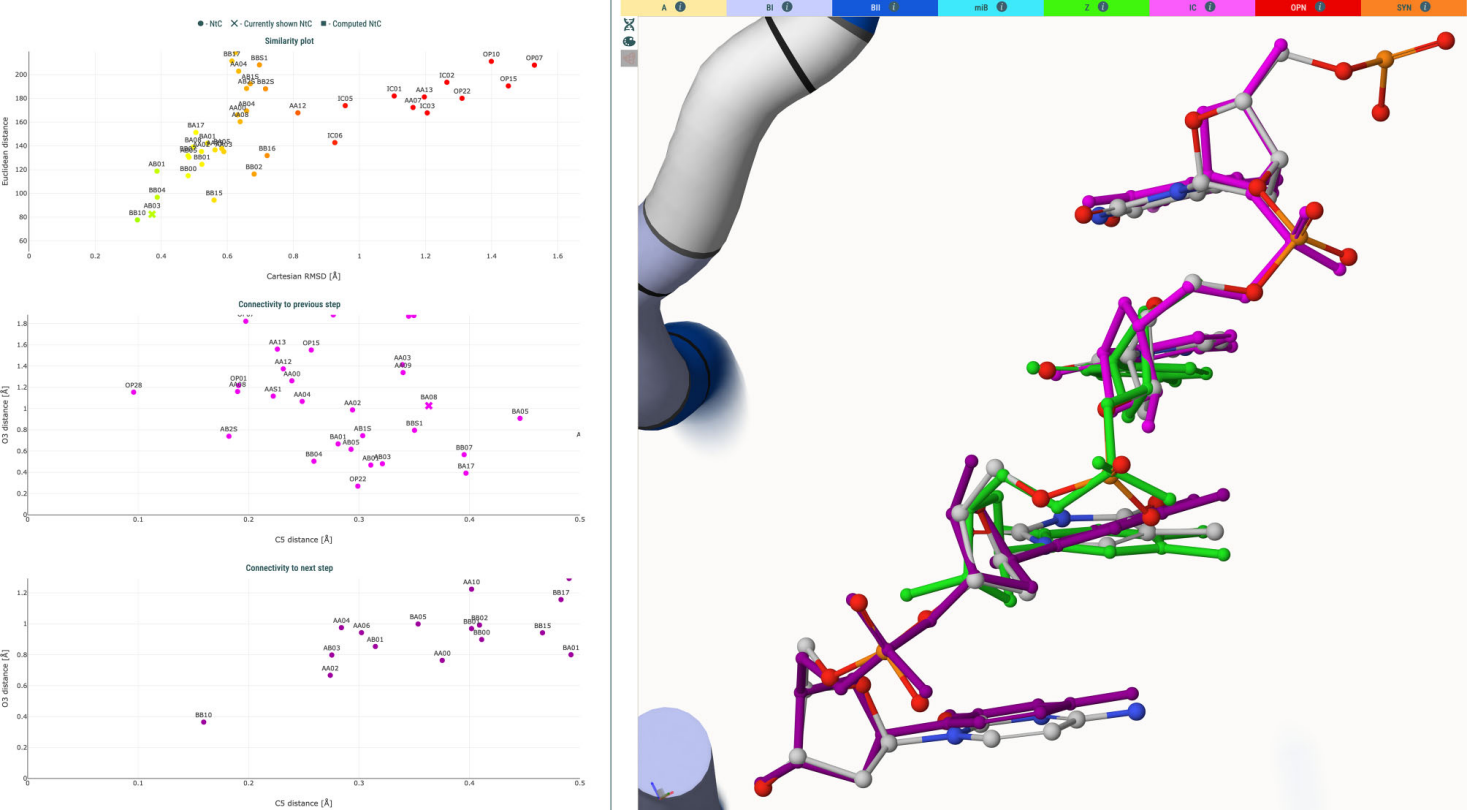
Similarity and connectivity plots for the C(–58)–T(–57) dinucleotide of chain J in Histone DNA (PDB 6jxd [58]). The experimentally determined dinucleotide is rendered as a ball-and-stick model colored by atom type, overlaid with the closest NtC conformer (BB10) in green. The previous (T(–59)–C(–58)) and next (T(–57)–C(–56)) dinucleotides are shown in magenta and dark purple, respectively.

DNATCO enables iterative improvement of nucleic-acid models by suggesting NtC classes compatible with both the central step and its flanking neighbors. After identifying candidate steps (typically those with NtC = NANT or those far from their assigned conformer), the user can preview the effects of adopting alternative NtC classes. Connectivity plots compare RMSD and torsional deviations of the edited step to its 5′ and 3′ neighbors, ensuring that modifications preserve chain continuity (Fig. 6).

In the nucleosome DNA, the C(–58)–T(–57) dinucleotide is a textbook refinement target: torsions lie between BB10 and AB03, and correcting the ζ and α angles brings the step closer to canonical B-form geometry without disturbing neighboring steps. DNATCO automatically produces torsion-restraint files for major refinement programs (Refmac [60, 61], Phenix [51], and BUSTER [62]) and for MacroMolecule Builder (MMB [63]), enabling seamless integration into refinement workflows.

#### Annotation of quadruplex structures

**Figure 7.**
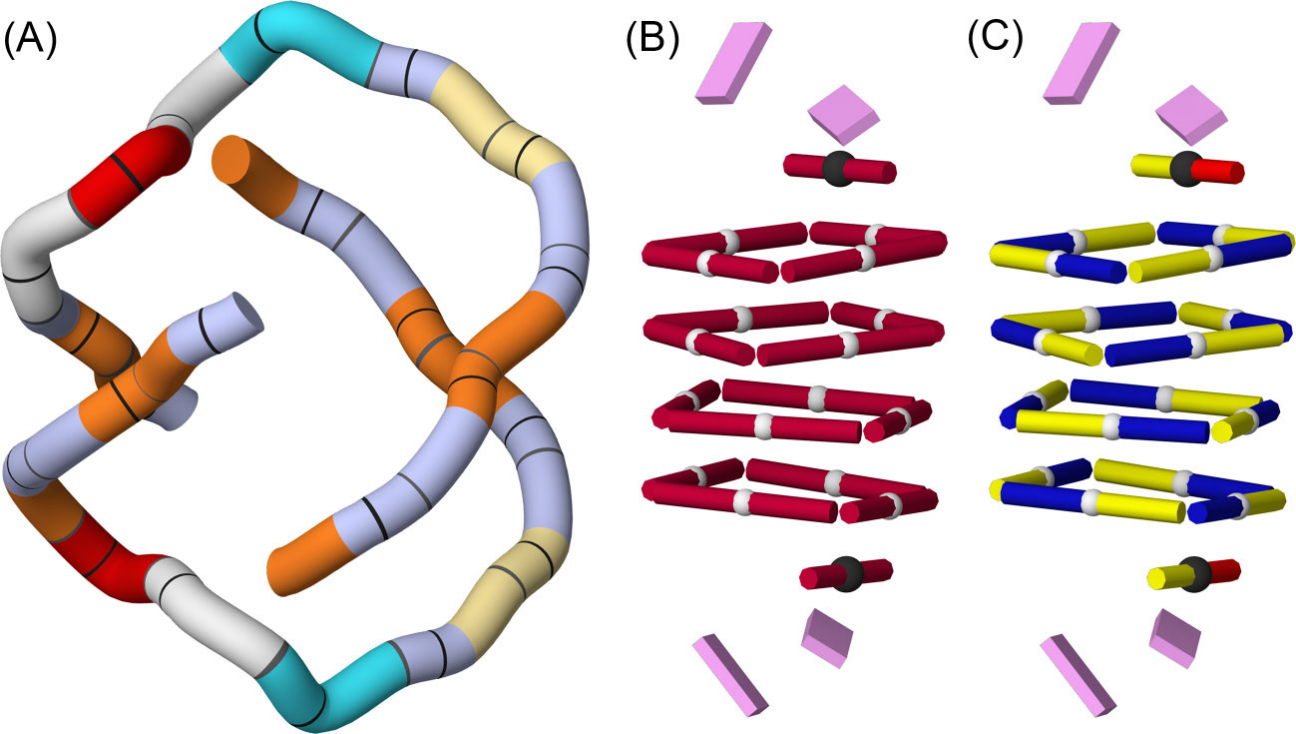
Visualization of structural features of Oxytricha quadruplex 1jpq [64]. (**A**) NtC-tube representation, color-mapped by dinucleotide conformation, showing the structural complexity of this structure. (**B**) Base-pair ladder view with non-canonical pairs in dark red. (**C**) Base-pair ladder view with color-coded base edges forming the pairs: yellow for the Watson–Crick edges, blue for Hoogsteen, and red for the sugar edges. *Cis*-oriented pairs are marked by white spheres, *trans*-oriented by black spheres, and unpaired bases by pink tiles.

Structures of quadruplexes such as 1jpq [64] and 5hix [65] can be described by the existing NtC classes, including characteristic quadruplex NtC classes such as BBS1 and BB2S, and visualization of the base pairs shows non-canonical pairing of the G-tetrads (Fig. 7). Color-coding of base pair classes distinguishes canonical (white tubes) and non-canonical pairs (red tubes) on the left but also provides more specific information on the interacting edges on the right side: the Watson–Crick edges are yellow, Hoogsteen blue, and sugar edge red. Visualization of both backbone as well as pairs is interactively linked to the tables of NtC conformers and base pairs.

Validation of valence geometry of this 1.6 Å high-resolution structure reveals 20 angles “Of Concern” mostly in bases. We can hypothesize that (some of these) angles are deformed by interactions with K^+^ cations that stabilize the tetraplex.

### Refinement guided by NtC similarity and connectivity

The similarity and connectivity plots described above to suggest refinement improvement of the histone-wrapped DNA in 6jxd [58] have been successfully applied to challenging systems, including DNA 18-mers with anisotropic diffraction [66, 67] and transcription-factor complexes 7bhy, 7oyk [68] and 8r3g, 8r7y [69]. Automated refinement via PDB-REDO [22] also improved 6jxd, raising its confal percentile, reducing unassigned steps, and lowering the excessive BII population—although database-wide trends show that automated pipelines do not always correct NA conformational issues, underscoring the value of targeted, NtC-guided refinement.

Supplementary Table S1 in the Supplementary Material compares the percentage of assigned NtC classes and the frequency of “Of Concern” distances and angles in structures taken directly from the PDB archival files versus those structures refined by PDB-REDO [22]. While refinement by PDB-REDO generally shows an improvement, the gain—more assigned NtC classes and fewer “Of Concern” valence geometries—is often marginal. This suggests a critical need for further improvements in refinement strategies that move beyond the scope of current PDB-REDO methods. For low-resolution experimental structures, a promising approach to achieve this improvement is the utilization of restraints based on the geometries of the DiNucleotide Conformers (NtC classes) during the refinement process. Examples of such DNATCO restraints are provided at the end of the supplement.

### Accessing outputs and broader NtC resources

For each analyzed structure, DNATCO provides downloadable mmCIF files enriched with NtC and valence-geometry annotations, CSV/JSON tables of conformers, and validation reports including RSCC–RMSD plots and detailed bond-geometry summaries. These exports are suitable for downstream analysis, refinement, and benchmarking.

For broader exploration, DNATCO’s integrated NtC and base-pair resources allow users to search PDB examples of each conformer, examine their torsion signatures, and review RSCC–RMSD distributions across resolutions. This supports comparative studies of canonical versus non-canonical backbones, tertiary-motif classification, and data-driven refinement strategies.

### Valence geometry validation

**Figure 8.**
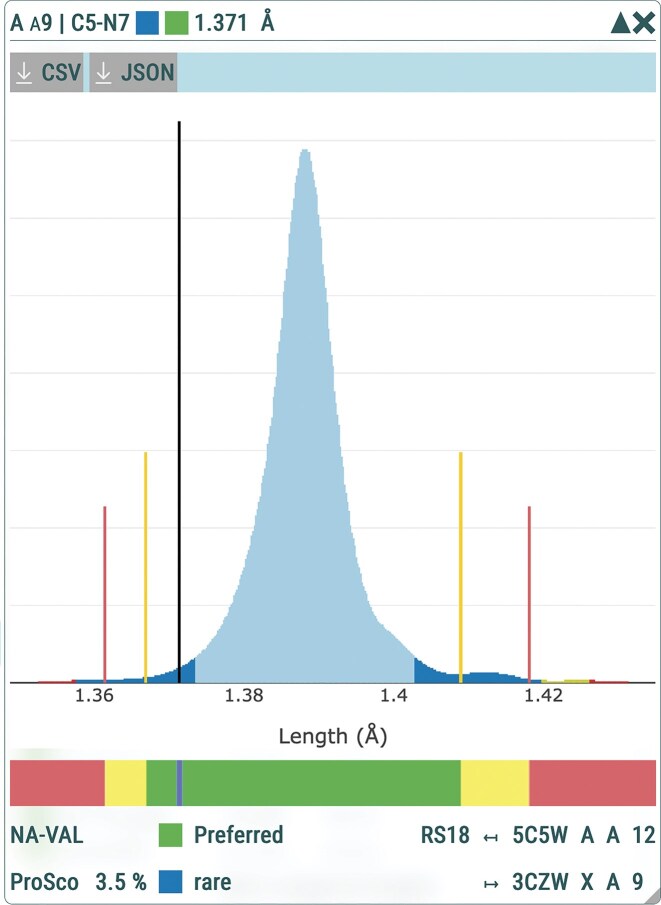
Example of NA-VAL bond length and bond angle validation for residue A9 in structure 1ehz [77]. Distribution of the C5–N7 bond length in the PDB-NA Reference Set (blue density) with the observed value for residue A9 (vertical black line). The NA-VAL color bar denotes the Preferred (green), Allowed (yellow), and Of Concern (red) intervals derived from combined CSD and reference-set statistics. For this bond, the Preferred region is widened by the CSD ± 3σ range, resulting in a larger green interval than would be obtained from PDB-NA Reference Set data alone. The observed value of 1.371 Å has a ProSco of 3.5%, placing it in the Rare category—unusual but still supported by a small number of reference-set observations.

For each bond and angle in a nucleic-acid structure, DNATCO v5.0 implements the three-tier valence-geometry classification according to [53] reporting Preferred, Allowed, and Of Concern tiers together with the underlying ProSco score, and the corresponding CSD-derived ranges (Fig. 8) [see the “Materials and methods” sections and [53] for more details]. This enables a detailed examination of unusual or suspicious geometries. The validation interface lists the corresponding residues from the PDB-NA Reference Set for any feature flagged as Of Concern, facilitating transparent inspection and contextual interpretation, in line with recommendation R4 of the Working Group on Nucleic Acid Valence Geometry.

To further improve interpretability—particularly in the context of structure refinement—each ProSco value is supplemented with an annotation describing how commonly the corresponding bond length or angle occurs within the PDB-NA Reference Set. These annotations classify every validated value as Common, Rare, Ambiguous, or Unique. This annotation is intended to complement the three-tier validation scheme by indicating how well each bond length or angle is supported by the empirical distribution of values available in the reference set used to analyze the valence geometries. In DNATCO, the four categories are visually encoded using shades of blue, from light (Common) to dark (Unique), providing an intuitive overview of geometric reliability. See Fig. 8 for illustrative examples. For bonds or angles with very low ProSco values, DNATCO lists the nearest matching cases from the PDB-NA Reference Set to encourage users to examine the structural context of these reference residues—including factors such as resolution, local environment, and experimental conditions—that may help explain or justify deviations from the most observed geometries.

### Reduced dimensionality representations of nucleic-acid geometry

Describing nucleic-acid conformation in full Cartesian detail is both impractical and uninformative for most analytical purposes, since it obscures recurring structural patterns within thousands of atoms. For this reason, a variety of reduced-dimensionality representations have been developed over the past several decades, each aiming to capture the essential aspects of NA geometry. Early approaches focused on base-pair and base-step parameters, treating nucleic acids primarily as helical assemblies of rigid bases. Subsequent methods introduced pseudo-torsional descriptors such as η/θ, offering compact, Ramachandran-like plots intended to summarize the overall shape of RNA backbones [70]. More recent frameworks, such as RNA backbone suites [46] or the NtC classes [47], attempt to classify sugar-to-sugar fragments using backbone torsions.

Base-pair and base-step parameters, as implemented in tools such as 3DNA [71] and DSSR [14], were originally designed to describe regular double helices by treating base pairs as rigid bodies arranged around an idealized helical axis. These descriptors work reliably for right-handed A- and B-form duplexes and their slightly deformed versions, but they become problematic in highly distorted or protein-bound regions where deviations from the B-form are. Under such conditions, base-step parameters often fluctuate, making them difficult or impossible to interpret in terms of meaningful conformational states. The limitations of purely helical or base-centric representations are particularly evident for RNA, where roughly half of the nucleotides in structured molecules are non-helical [14].

For example, in several DNA–protein complexes (PDB IDs 1a73 [72], 4r2a [73], 6q1v [74]), >40% of nucleotides at the protein interface adopt A-like conformations, often forming extended A-form stretches rather than isolated perturbations. These mixed or transitional B-A geometries expose weaknesses in base-parameter classifications. Such inconsistencies illustrate that base-derived classifiers are highly sensitive to local distortions in base orientation or stacking and therefore fail to capture the true conformational diversity arising in functional environments where backbone geometry, sugar pucker, and χ torsion are tightly coupled.

Molecular conformations are traditionally described by torsion angles. Early studies correlating a few torsions, typically two, helped to discover e.g. BI and BII forms [75] but failed to capture the conformational diversity of large NA segments, especially in RNA. In early 2000, it became clear that the smallest unit that provides a more comprehensive description of NA conformations must contain a sugar-to-sugar unit linked by the phosphodiester linkage in the middle [43–45]. Later developed “consensus RNA conformers” [46] and the related RNA backbone suites provide a systematic classification of phosphodiester geometry but without consideration of the sugar-base orientation described by torsion χ at the glycosidic bond. Inclusion of these two critical degrees of freedom and explicit consideration of the base–base distances in the sugar-to-sugar near dinucleotide unit led to the formulation of universal dinucleotide conformational classes valid for both RNA and DNA [47], which are one of the cornerstones of the DNATCO annotation.

The “Ramachandran-like” two-dimensional η/θ pseudo-torsion representation offers an even greater dimensionality reduction by representing the local trinucleotide geometry by two pseudo-torsion angles assigned to each central nucleotide [70, 76]. Although η/θ plots reveal broad regions associated with helical and non-helical states, many distinct combinations of backbone torsions, χ values, and sugar puckers project onto the same region of pseudo-torsional space, causing helical and non-helical conformers to overlap. The NtC-based RNA analysis shows that a wide variety of non-helical geometries, including strongly perturbed, unstacked conformers characterized as “open” dinucleotides, NtC classes OPxx, fall directly within the central η/θ region commonly interpreted as helical.

**Figure 9.**
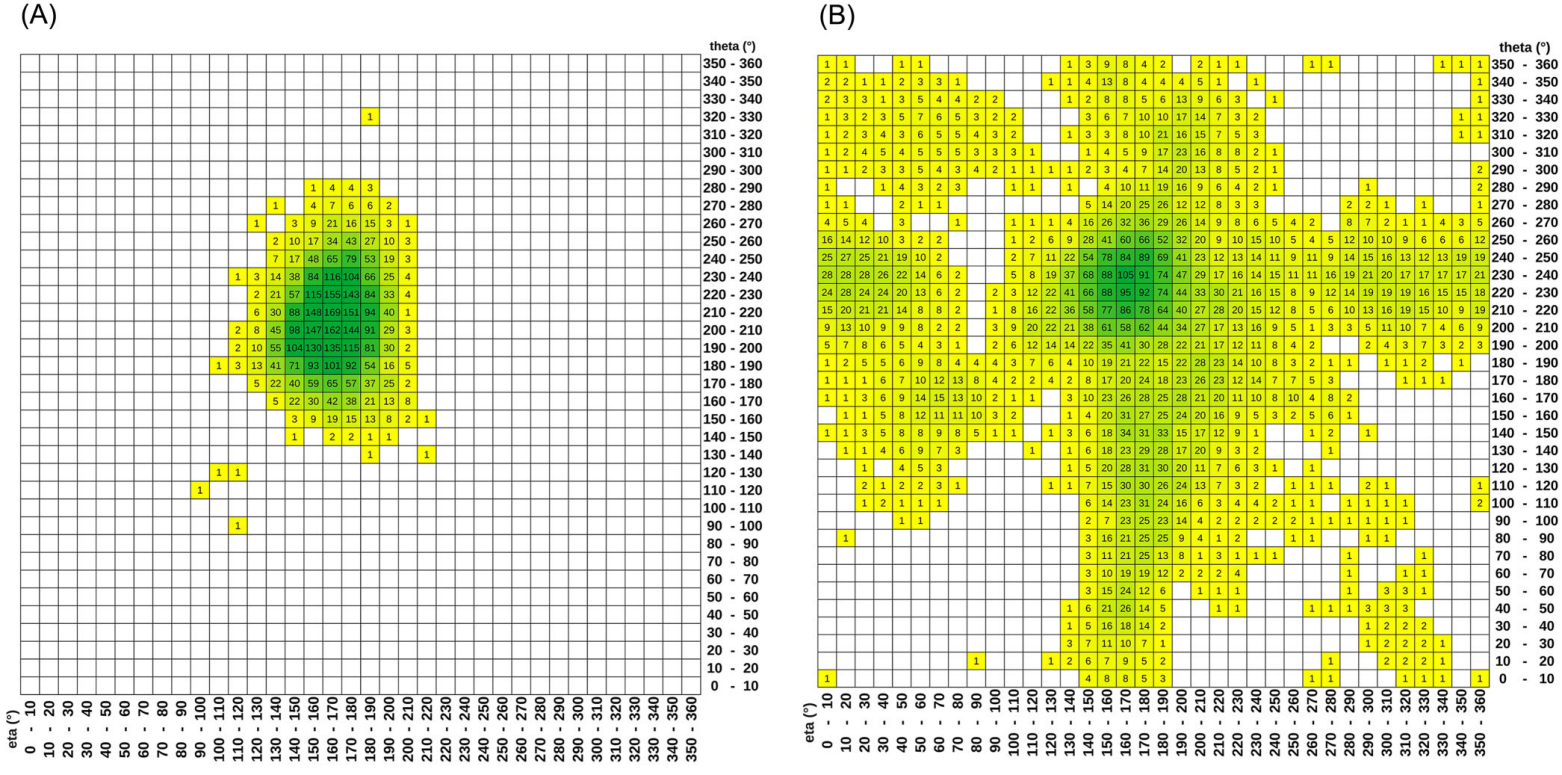
Distribution of NtC-assigned trinucleotide geometries in η/θ pseudo-torsion space. The heatmap shows the projection of “helical” trinucleotide RNA fragments from the PDB onto η/θ space of two overlapping dinucleotide steps. (**A**) The map shows RNA trinucleotides, which can be described as double helical, formed by the canonical Watson–Crick G–C or A–U pairs, while (**B**) the map contains non-helical regions of trinucleotides with at least one dinucleotide in OPxx conformation. Only trinucleotides in which both steps can be assigned to an NtC class with RMSD <0.5 Å to the class representative were used.

Projection of NtC-assigned RNA trinucleotide geometries into the η/θ pseudo-torsion space shows that η/θ blurs the distinction between helical and non-helical conformations (Fig. 9). Notably, many of these trinucleotides, which contain an OPxx conformer and cannot be a part of a double helix, map into the same or very similar region of the η/θ map. Therefore, two-dimensional η/θ NA geometry cannot support all-atom nucleic-acid reconstruction and serve as a foundation for nucleic-acid structure prediction and accurately capture the rich diversity of nucleic-acid structures across duplexes, loops, bulges, junctions, protein-induced deformations, and high-order architectures such as quadruplexes.

## Conclusions

We have presented DNATCO v5.0, a fully integrated web application (dnatco.datmos.org) for the annotation, validation, and refinement of nucleic-acid structures. DNATCO v5.0 supports both public and user-supplied models in mmCIF or PDB format and features a redesigned, TypeScript-powered React interface that caters to both experts and newcomers in structural biology.

At its core, DNATCO assigns each dinucleotide to one of 96 NtC conformer classes—organized within the CANA schema—and now also tabulates and visualizes base-pair topologies using the Leontis–Westhof nomenclature. Canonical G–C and A–U/T pairs render as white rods, while noncanonical pairs appear in dark red or, in the detailed scheme, are colored by interacting edges (Watson–Crick, Hoogsteen, sugar). This dual, color-coded representation of backbone and base-pair geometry delivers an intuitive view of key structural motifs.

DNATCO v5.0 further provides comprehensive valence-geometry validation (bond lengths and angles) against a curated PDB-NA Reference Set, along with RSCC/RMSD plots to highlight discrepancies between model and electron density. When potential issues arise, users can experiment with custom NtC definitions in the Refinement page—visualizing changes via similarity/connectivity plots—and export restraint files for downstream refinement in programs such as Refmac [60, 61], Phenix [51], and Buster [62], or input for (re)modeling a structure with the MMB [63].

All analysis steps (Annotation, Validation, Refinement) produce downloadable outputs in multiple formats—including an mmCIF enriched with DNATCO-specific categories—backed by context-sensitive help. By combining WebAssembly-accelerated local processing with server-side data services, DNATCO v5.0 offers a powerful, accessible platform that we anticipate will enhance the accuracy and reliability of nucleic-acid structural models and advance research across structural biology and bioinformatics.

## Supplementary Material

gkaf1491_Supplemental_File

## Data Availability

All data supporting this study are included within the manuscript and its Supplementary Material. The DNATCO-specific mmCIF dictionary (mmcif_ndb_ntc.dic) is available from the RCSB mmCIF repository (http://mmcif.rcsb.org/dictionaries/mmcif_ndb_ntc.dic/Index/). All source codes for DNATCO, including the submodules, the libLLKA C++ core structure–processing library, and accompanying CLI/GUI example programs, are hosted on GitHub (https://github.com/cernylab) under the MIT License. Datasets defining NtC classes for annotation and validation are provided in the same repository under a CC BY-SA 4.0 license. The fully offline multi-platform CLI version of the dnatco.datmos.org using Node.js is provided in the “dnatco.zip” file from the Zenodo repository (DOI: 10.5281/zenodo.14150199). Supplementary Data—including DNATCO-annotated mmCIF files, complete validation reports, and NtC-specific restraint files for PDB entries 1ehz [77], 4qvi [55], and 5hix [65]—are available in the Zenodo repository (DOI: 10.5281/zenodo.14150199).
