# Supplementary material for: DNATCO v5.0: integrated web platform for 3D nucleic acid structure analysis
Source: Nucleic Acids Res. 2026 Jan 6;54(1):gkaf1491. doi: 10.1093/nar/gkaf1491 (PMC12774647; doi:10.1093/nar/gkaf1491)
Supplement: gkaf1491_Supplemental_File [file gkaf1491_supplemental_file.pdf]

Supplementary material for

DNATCO v5.0: Integrated Web Platform for 3D Nucleic Acid Structure Analysis

Jiří Černý, Michal Malý, Paulína Božíková, Terezie Prchalová, Jakub Svoboda, Lada Biedermannová, and Bohdan Schneider *

Institute of Biotechnology of the Czech Academy of Sciences,
Průmyslová 595, Vestec, Czech Republic,

* Correspondence e-mail: bohdan.schneider@ibt.cas.cz

Detailed Architecture of the DNATCO Web Application

Figure S1 presents an expanded schematic of DNATCO's modular web architecture, highlighting the client-side C++ core (compiled to WebAssembly), the JavaScript/HTML user interface layers, and optional server-side services (e.g., MAXIT integration). The dependencies illustrated here are representative; for the complete list of libraries, packages, and tools, please visit dnatco.datmos.org.

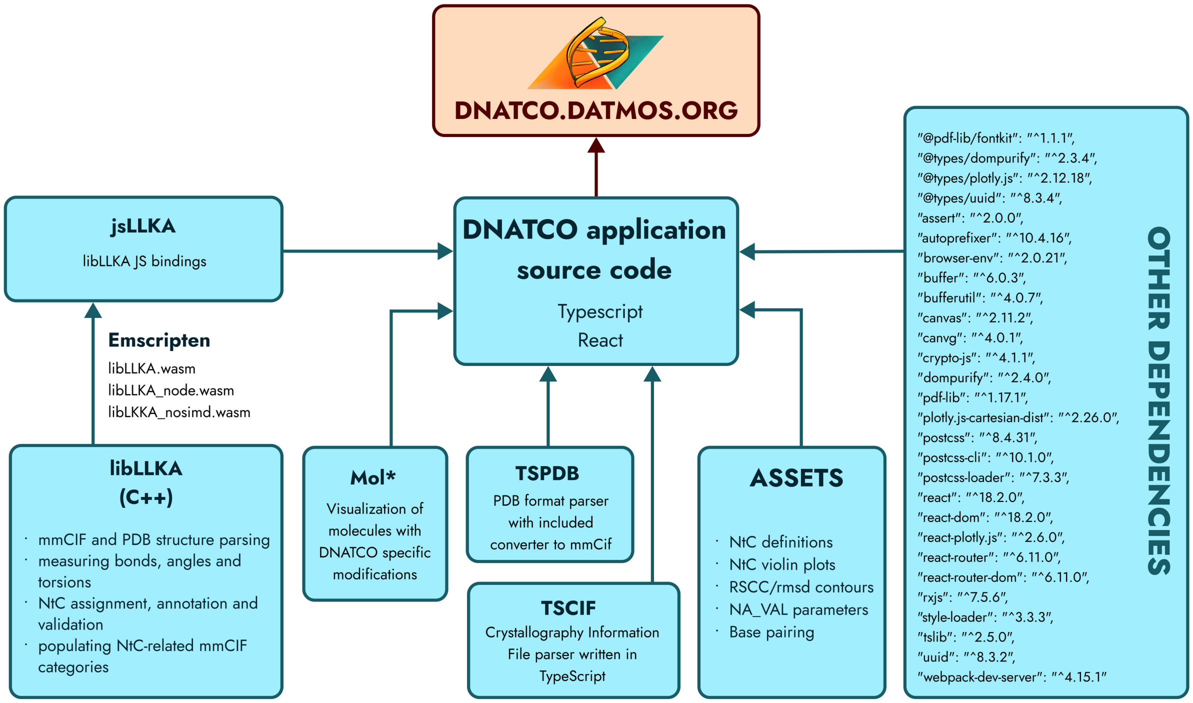

Figure S1. Detailed schematic representation of DNATCO's architecture.

A walkthrough of DNATCO modules

Home Page

The Home Page (Figure S2) is the starting point for DNATCO analyses, offering flexible input and clear next-step navigation:

- **Structure Retrieval**: Fetch any PDB entry by its ID from RCSB PDB (1,2)or PDB-REDO (3,4).
- **Custom Uploads**: Upload your own models in mmCIF or legacy PDB format to accommodate nonstandard or unpublished structures.
- **Density Maps**: Optionally include Fo–Fc, 2Fo–Fc, EM maps, or map coefficients; available maps for PDB entries load automatically.
- **Next Steps**: After input, choose **Annotation**, **Validation**, or **Refinement** to assign NtC conformers, evaluate structural quality, or generate torsion-restraint sets.

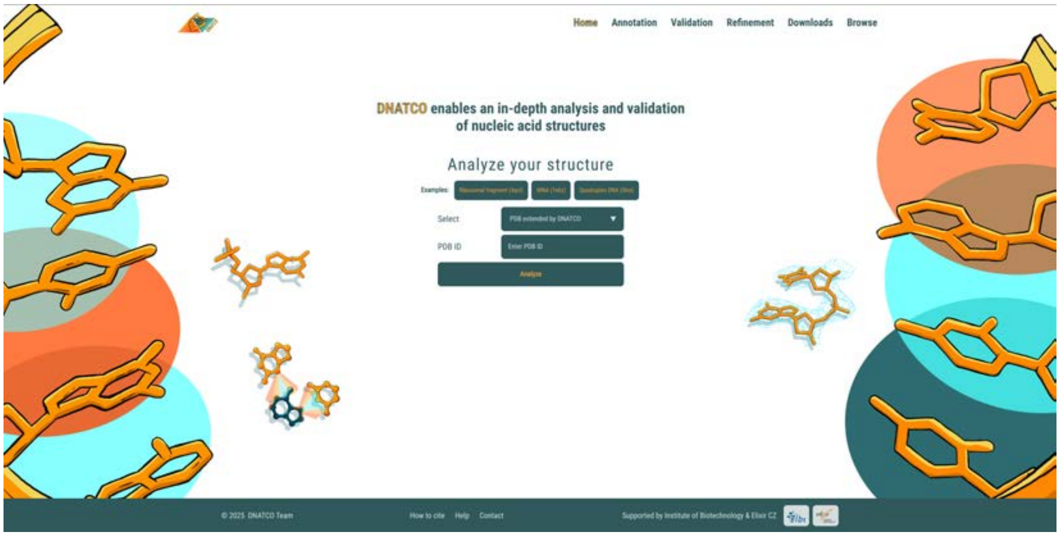

Figure S2. DNATCO Home page: users are presented with sample structures and can choose a new structure for analysis by entering its PDB code or uploading a custom coordinate file alongside the corresponding electron density maps (optional).

Annotation Page

The Annotation page offers a concise summary of the analysis, organized into five tabs along the left sidebar:

- **Conformation**
  Details each dinucleotide step's conformational assignment (NtC and CANA).

- **Base Pairs**
  Leontis–Westhof annotation of base pairs by edge (Watson–Crick, Hoogsteen, Sugar) and orientation (cis/trans).

- **Structure Info**
  Displays metadata and experimental parameters for the structure.

- **Downloads**
  Offers links to export coordinate files, annotation tables.

- **Help**
  Provides guidance on interpreting the annotations and using the interface.

On the ***Conformation tab*** (Figure S3), you'll find a table listing every detected dinucleotide step along with its assigned NtC class and CANA letter. Steps that could not be confidently assigned (labeled NANT) appear in a lighter font, and hovering over any NANT row reveals the nearest NtC class as a tooltip.

To the right, the **Mol* viewer** shows the nucleic-acid structure in a simplified "tube" representation, colored according to our in-house NtC scheme. Above the model are three controls for toggling molecular representations, adjusting color schemes, and refining density display. A color bar at the top maps each hue to a conformer family, offering an immediate visual summary.

The table and 3D view are fully linked: clicking a row in the table highlights the corresponding step in the Mol* viewer, and selecting a tube segment in Mol* selects its entry in the table. Mol* also overlays the reference conformation (in green) on the selected step. For large assemblies—such as ribosomal complexes—you can filter the results to a single chain; the table will then list only that chain's steps, and Mol* will display only its coordinates.

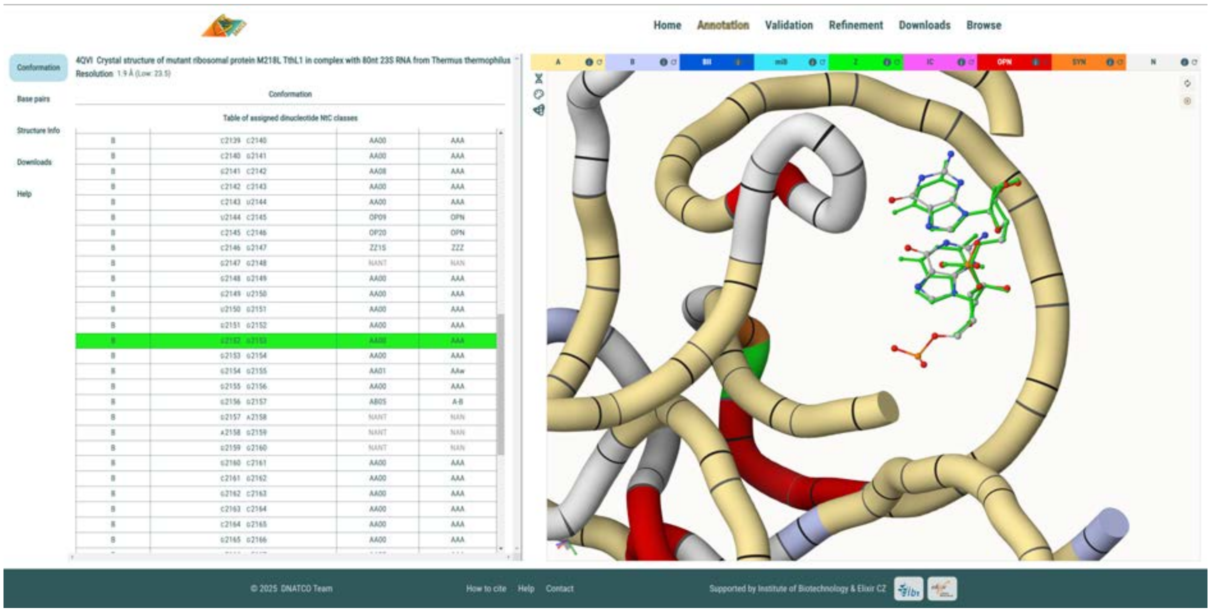

Figure S3. The ***Conformation*** tab with interconnected Mol* viewer functionality. The selected dinucleotide is shown in stick representation alongside its NtC reference (green). This example uses PDB entry 4qvi - an 80-nt fragment of 23S rRNA (5). An interactive

version is available at https://dnatco.datmos.org/app/dnatco/annotation/assigned-ntcs?cifcode=4qvi&stepName=4qvi_B_U_2109_G_2110.

The *Base Pairs* tab presents base-pair classifications as determined by the FR3D program (6). In the 4QVI structure, just over half of the detected base pairs are canonical Watson–Crick pairs; the remainder are non-canonical pairs that also contribute significantly to the molecule's stability.
The *Structure Info* is where you'll find all metadata extracted from the mmCIF file—experimental method, resolution, deposition date, and literature citation—followed by a **Main Features** section with summary tables. The Counts of NtC table in this section shows that roughly two-thirds of the dinucleotide steps in the example 4qvi structure adopt A-like conformers, about one-sixth are unassigned (reflecting either incomplete refinement or rare geometries), and the rest include a small B-like population plus more complex folds such as open (OPN), intercalated (IC), and left-handed Z-like (Z) conformations.

In the same section, the **Counts of Nucleotides in Polymer Entities** table summarizes the structure's nucleotide composition. For PDB entry 4qvi, it reveals a G-rich sequence comprised exclusively of the four standard bases (G, A, U, C). When modified nucleotides are present, DNATCO currently recognizes 559 non-standard residues: 401 of these share identical atomic connectivity and naming with their standard counterparts, while the remaining 158 are included via explicit atom-name mappings. All supported non-standard residues are counted alongside the standard bases in this table.

The ***Downloads*** tab offers links to export the assigned NtC table in CSV or JSON formats, as well as an annotated mmCIF file containing detailed NtC assignment data.
The ***Help*** tab provides definitions of NtC and CANA letters, a glossary of key terms, and user guides to aid in interpreting the analyses.

Both tabs appear on every page, with their specific download files and help content tailored to the context of each page.

Validation Page

This page uses the same tabbed layout as the Annotation page but delivers a far more detailed analysis of structural features. It functions as a comprehensive toolbox for both valence-geometry and conformational validation.
The *Overall Quality* tab (Figure S4) presents two key validation metrics:

1. **Backbone conformational quality**
    - **NtC assignments & confal scores (CS):** Quantify how well each dinucleotide step matches its assigned NtC class. Scores range from 0 (no match) to 100 (perfect match).
    - **RMSD to Golden Set references:** Calculated between the analyzed step and the class representative. Comparison is based on the same 18 atoms that define the backbone torsions—from C5′ of nucleotide n through O3′ of nucleotide n + 1—plus the O4′, C1′, N1/N9, and C2/C4 atoms of both residues.
2. **Valence geometry quality**

- Compares bond lengths and angle measurements in your structure against reference distributions derived from the PDB Reference Set.

- **Color bars** indicate the fraction of values with ProSco scores ≥ 5 (green), 0.01–5 (yellow), and < 0.01 (red).

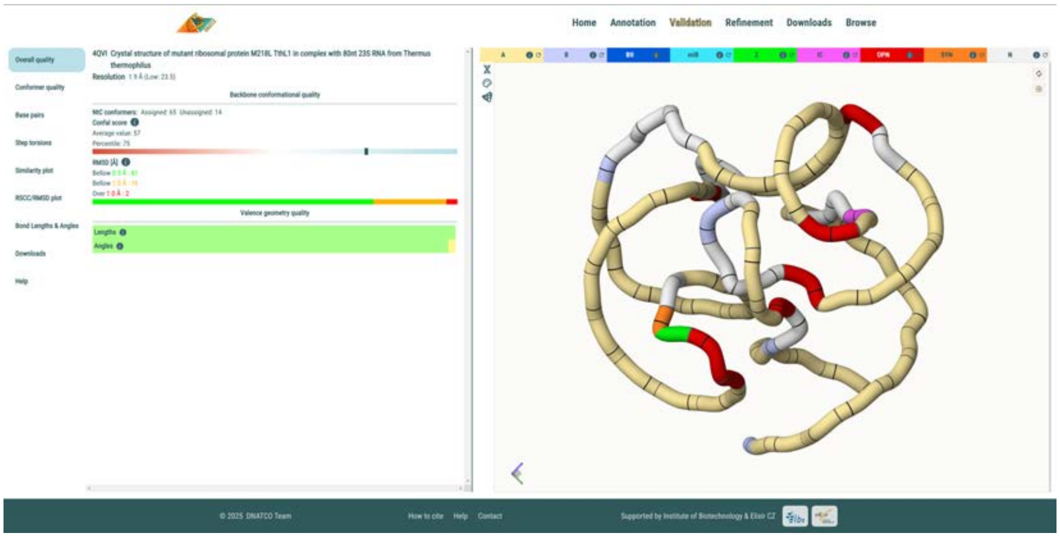

Figure S4. ***Overall Quality*** tab on the Validation page, shown for the 80-nt 23S RNA from PDB entry 4qvi {Tishchenko, 2015 #6777}. An interactive version is available at https://dnatco.datmos.org/app/dnatco/validation/overall-quality?cifcode=4qvi.

The *Conformer Quality* tab displays a table of all dinucleotide steps with their assigned NtC classes, CANA letters, and corresponding confal and RMSD scores. Each score is color-coded from red (poor) through yellow to green (excellent), providing an at-a-glance visual indicator of conformational quality. **The *Base Pairs* tab s**ummarizes base-pair classifications as determined by the FR3D program {Sarver, 2008 #6910}. This mirrors the Base Pairs tab found on the Annotation page.

The *Step Torsions* tab shows the raw NtC torsion angles for a selected dinucleotide step alongside reference values. The plot overlays the step's values (black line) on violin plots representing the distribution of these parameters in experimental structures {Cerny, 2020 #6819}.

The ***Similarity Plot*** tab (Figure S5) features an interactive scatter plot comparing the selected dinucleotide step to all 96 NtC reference classes by Cartesian RMSD versus torsional-space Euclidean distance. Each point is colored on a "semaphore" scale by its RMSD:

- **Red** for values > 1.0 Å

- **Yellow–green** for values ≲ 0.5 Å

Points in the green-to-yellow range (RMSD ≲ 0.5 Å) indicate conformers that are sufficiently similar in Cartesian space to serve as reasonable alternative models during further structure refinement.

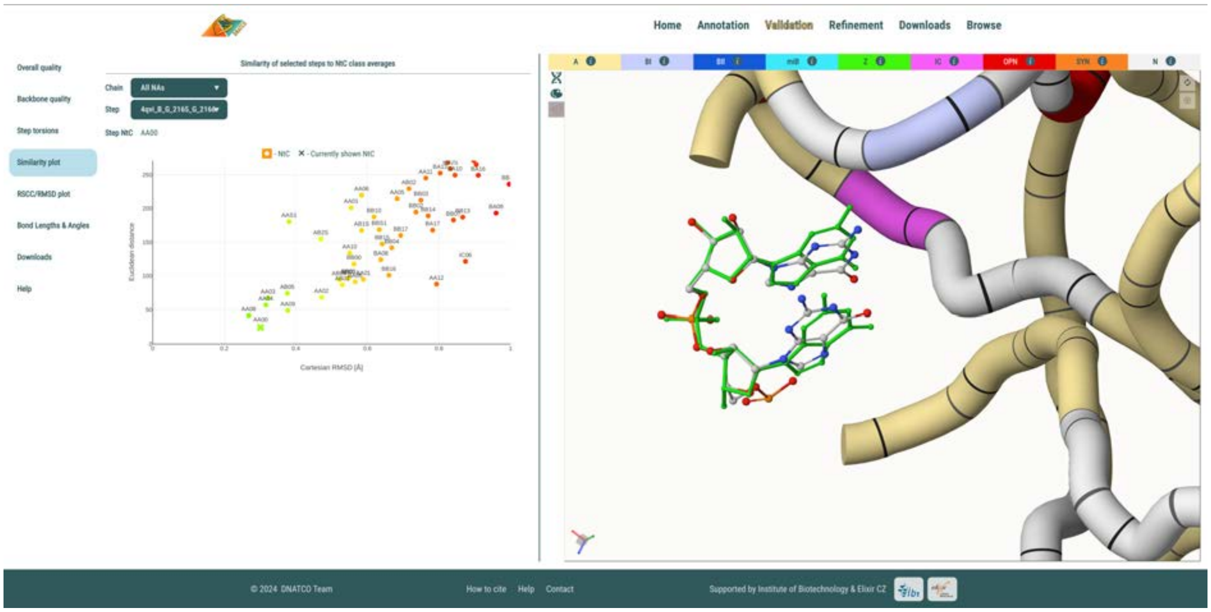

Figure S5. Similarity plot for dinucleotide step G_2120–G_2121 in chain B of PDB entry 4QVI {Tishchenko, 2015 #6777}. An interactive version is available at https://dnatco.datmos.org/app/dnatco/validation/similarity-plot?cifcode=4qvi&stepName=4qvi_B_G_2120_G_2121.

The ***RSCC/RMSD Plot*** tab (Figure S6) features two interactive scatter plots—one for steps with assigned NtC classes and one for unassigned steps—each showing real-space correlation coefficient (RSCC) versus RMSD. Both are overlaid on a contour map representing the full-structure dataset. This dual view lets you evaluate each dinucleotide's local quality by combining its fit to the electron density (RSCC) with its structural deviation (RMSD). Clicking any point in either plot will select that dinucleotide in the Mol* viewer.

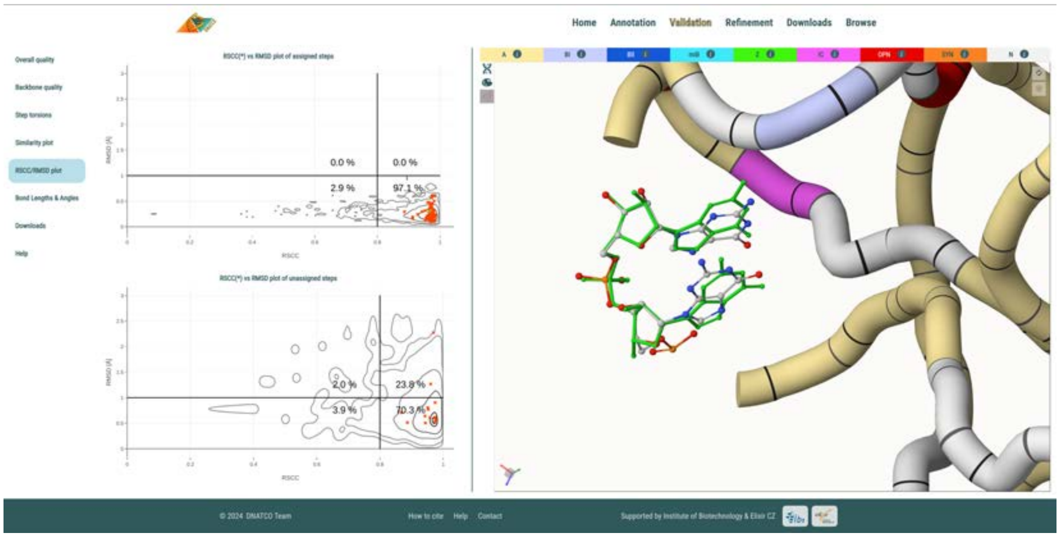

Figure S6. RSCC/RMSD tab on the Validation page, showing scatter plots for assigned (top) and unassigned (bottom) dinucleotides from PDB entry 4qvi {Tishchenko, 2015 #6777}. An

interactive version is available at https://dnatco.datmos.org/app/dnatco/validation/rscc-plot?cifcode=4qvi.

The **Bond Lengths & Angles** tab (Figure S7) shifts the focus from dinucleotide conformers to the geometry of individual nucleotides. For the entire structure—or any selected chain—each bond length and angle is compared against the distribution of values in the curated PDB Reference Set. Horizontal stripes, color-coded by ProSco score (green = 5–100; yellow = 0.01–5; red < 0.01), indicate the fraction of parameters falling into each range.
At the top, you'll see overall valence-geometry statistics for your chosen scope, followed by an expandable list of individual residues. Clicking **Lengths** or **Angles** (abbreviated **L** and **A** for residues) opens popup summary tables. You can then expand any residue to view its specific bond-length and angle values alongside their probabilities. Clicking the colored stripe beside a residue launches a graph that overlays the observed parameter on the reference distribution. Finally, an additional section—also expandable—lists the least probable (worst) bond-length and angle values.

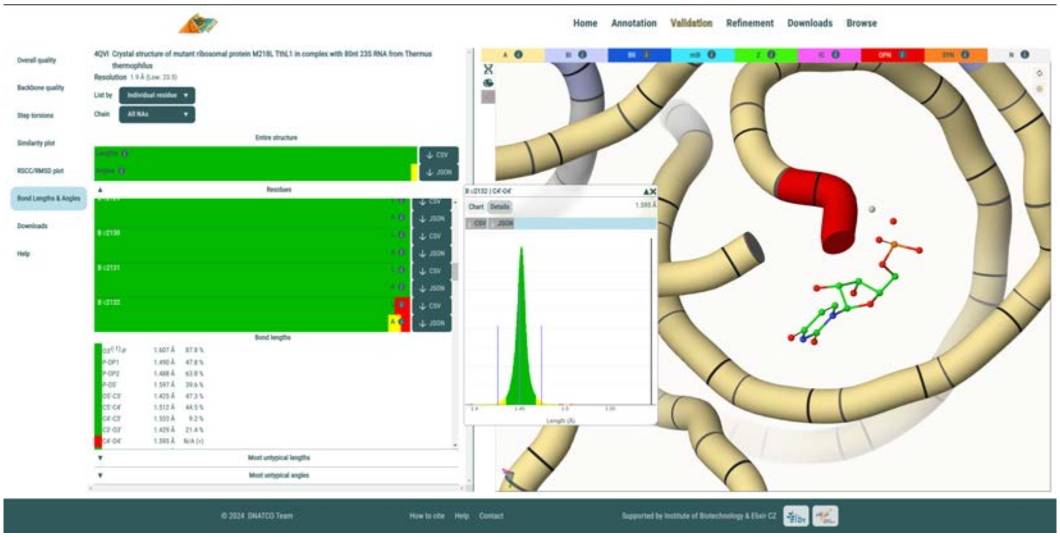

Figure S7. ***Bond Lengths & Angles*** tab showing the C4'–O4' bond in U28 from PDB entry 4qvi {Tishchenko, 2015 #6777}. The popup window displays a color-coded ProSco distribution of C4'–O4' bond lengths extracted from the curated high-resolution PDB Reference Set (see Methods). Blue lines mark the mean ± 3σ of the corresponding CSD-derived distribution {Kowiel, 2020 #6823; Kowiel, 2016 #6824}, while the black line at 1.595 Å indicates the observed value. In this case, the measured bond is significantly longer than those in both the CSD and the PDB Reference Set. An interactive version is available at https://dnatco.datmos.org/app/dnatco/validation/angles-lengths?cifcode=1ehz.

The ***Downloads*** tab provides additional validation files for download:

- **Bond Lengths, Bond Angles & Geometry** reports
- **RSCC/RMSD** plots (SVG format)

- **DNATCO Structure Validation Report** (PDF or plain-text)

Refinement Page

The **Refinement page** lets users adjust NtC assignments and immediately review the effects—both visually and via similarity and connectivity plots. You can create and compare multiple custom NtC sets for downstream structure refinement. Note that the default "(Computed)" set, generated automatically by DNATCO, is read-only and cannot be altered.

Alongside the similarity plot (also available in Validation), t**he *Connectivity Plot*** tab provides two connectivity plots (Figure S8) that visualize the chemical links of the central dinucleotide within the polymer chain. Because modifying a step can alter its C5′ bond to the preceding nucleotide and its O3′ bond to the following one, these plots help verify proper linkage:

- **Previous-step connectivity:** Plots the distances between the C5′ and O3′ atoms of the first residue in the selected step and the C5′ and O3′ atoms of the second residue in the immediately preceding step.
- **Next-step connectivity:** Plots the distances between the C5′ and O3′ atoms of the second residue in the selected step and the C5′ and O3′ atoms of the first residue in the next step.

The *Change NtCs* tab lets you manually override NtC assignments. You can create and save multiple custom sets of dinucleotide conformers, which can then be exported as restraint files for downstream refinement in structural-modeling packages.

MMB commands file, MMB will try to force the structure geometry into the prescribed NtCs.

The ***REFMAC Restraints*** tab generates a list of geometric restraints—derived from your current NtC set—for use with REFMAC {Murshudov, 1996 #3920; Agirre, 2023 #6912}. An excerpt from the auto-generated REFMAC restraint file is included in the supplementary material.
The ***Phenix Restraints*** tab works identically but produces restraints formatted for PHENIX {Liebschner, 2019 #6828}.

The ***Buster Restraints*** tab likewise exports restraints for BUSTER {Roversi, 2000 #6914}. Examples of each restraint file format appear in the supplementary material.

Finally, the ***MMB Commands File*** tab provides a snippet of MacroMolecule Builder (MMB) commands {Flores, 2010 #6913}. Adding these commands to an MMB script will enforce your prescribed NtC conformations during model building.

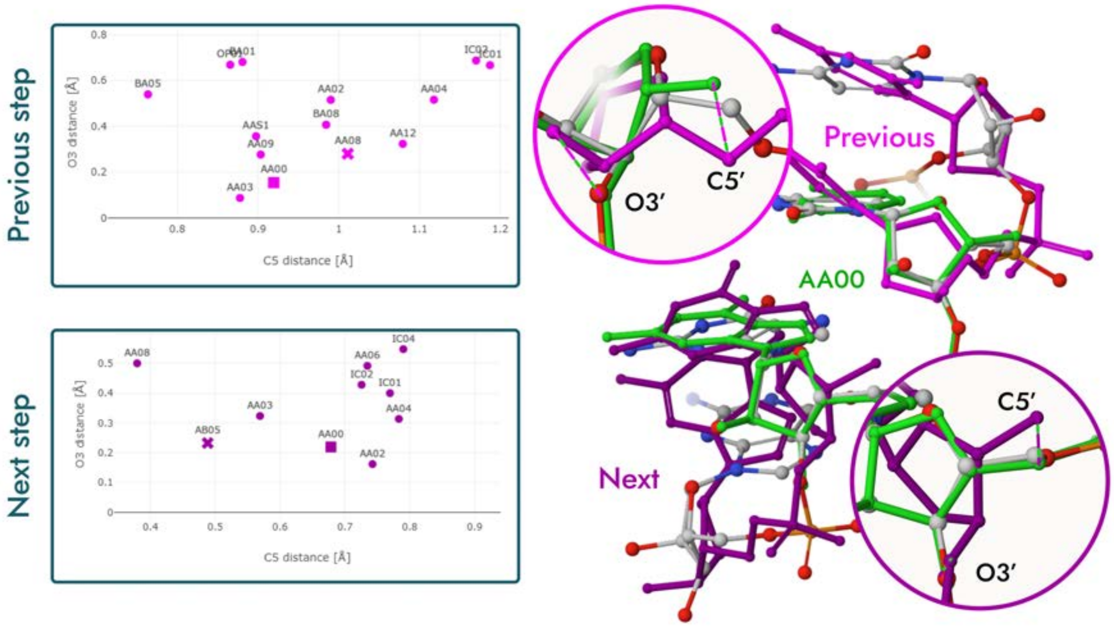

Figure S8. Connectivity plots for dinucleotide C 2164–G 2165 in chain B of PDB entry 4qvi {Tishchenko, 2015 #6777}, showing links to the preceding (left) and following (right) steps. Squares denote the best-calculated NtC assignment, while the currently selected NtC is marked with an "X." Here, the chosen conformation fails to connect properly to either neighbor. An interactive version is available at https://dnatco.datmos.org/app/dnatco/refinement/connectivity-plot?cifcode=4qvi&stepName=4qvi_B_C_2164_G_2165.

Downloads Page

The Downloads page provides access to all annotation and validation data for a selected PDB structure or user-uploaded model. Key files include:

- **Extended mmCIF**
  The primary DNATCO output: the original mmCIF file augmented with DNATCO-specific categories, containing the full annotation and validation of nucleic acid conformations.
- **Conformer Annotation Tables**
  Assigned NtC tables in both CSV and JSON formats, available as:
  - **Simplified** – basic assignment information
  - **Full** – includes all validation metrics (confal scores, RMSDs, etc.)
- **Valence Geometry Reports**
  Measured bond-length and bond-angle lists for backbone and base atoms in standard nucleotides, downloadable in CSV or JSON. Reports are provided:
  - **Per-residue** – detailed statistics for each nucleotide
  - **By nucleotide type** – aggregated data to identify systematic trends
- **RSCC/RMSD Plots**
  Real-space correlation versus RMSD scatter plots in SVG format.
- **Structure Validation Report**
  A comprehensive DNATCO validation summary, available as PDF or plain-text.

All files can be downloaded directly from this page for streamlined integration into downstream analysis and refinement workflows.

Browse Page

The Browse page offers a comprehensive overview of NtC dinucleotide conformers and their occurrences in the PDB. The ***Conformers*** **tab enables to s**earch the PDB for examples of specific NtC steps. Results appear in a table with columns for step name, NtC class, CANA category, confal score, RMSD, resolution, and electron density availability. Clicking a step name opens a new browser tab featuring the DNATCO analysis for that structure and highlights the selected dinucleotide in the 3D view. The ***Base Pairs*** **tab o**pens the basepairs.datmos.org resource in a new tab for detailed base-pair classifications. The ***Table of Conformers*** **tab l**ists all 96 NtC classes alongside their annotations, frequencies in DNA and RNA structures, and defining torsion-angle parameters. The ***Contour Plots*** **tab d**isplays interactive contour plots for each NtC class in RSCC versus RMSD space, enabling visual inspection of conformer distributions.

Comparison of PDB-archived and PDB-REDO re-refined structure features

Table S1. Structure statistics for X-ray crystal models of DNA and RNA from the PDB archive (PDB columns) versus their PDB-REDO refinements (3).

	resolution range (Å)[1]		# steps[2]	% of NANT[4] PDB-REDO	PDB
DNA	0.0	1.8	28,745	15.5	15.2
	1.8	2.4	86,564	14.4	15
	2.4	3.0	116,649	19.5	20.3
	3.0	<	79,645	28.9	28.8
RNA	0.0	1.8	11,942	15.1	14.3
	1.8	2.4	70,480	22.5	22.1
	2.4	3.0	218,825	24.8	24
	3.0	<	321,308	29.3	28

	resolution range (Å)[1]		# bonds[3]	% of concern - bonds[5] PDB-REDO	PDB
DNA	0.0	1.8	655 k	0.15	1.1
	1.8	2.4	2.0 M	0.05	0.4
	2.4	3.0	2.6 M	0.11	0.24
	3.0	<	1.8 M	0.05	0.24
RNA	0.0	1.8	252 k	0.11	0.55
	1.8	2.4	1.3 M	0.03	0.26
	2.4	3.0	3.8 M	0.02	0.09
	3.0	<	7.3 M	0.06	0.25

				% of concern - angles[5]	
	resolution range (Å)[1]		# angles[3]	PDB-REDO	PDB
DNA	0.0	1.8	1.0 M	0.43	1.19
	1.8	2.4	3.1 M	0.45	0.6
	2.4	3.0	4.0 M	0.57	0.41
	3.0	<	2.8 M	0.55	0.34
RNA	0.0	1.8	390 k	0.43	0.43
	1.8	2.4	2.1 M	0.46	0.33
	2.4	3.0	5.9 M	0.56	0.24
	3.0	<	11.4 M	0.91	0.62

[1] Range of crystallographic resolutions for the selected structures. [2] Total number of dinucleotide steps analyzed. [3] Total counts of backbone bond lengths / bond angles. [4] Percentage of dinucleotides unassigned to any NtC class (formally class NANT). [5] Percentages of bond lengths and bond angles flagged as “of concern,” indicating outlier geometry (7).

DNATCO Restraints

Excerpts from automatically generated restraint files for REFMAC (8,9) , Buster (10) and for Phenix (11). These restraints target the δ1 torsion—defined by ribose atoms C5′, C4′, C3′, and O3′ (see dnatco.datmos.org/app/about for the dinucleotide fragment definition)—for the step G_2105–G_2106 in the 23S rRNA of PDB entry 4qvi (12).

REFMAC restraints snippet:

```
external torsion first chain B residue 2105 atom C5'
             next chain B residue 2105 atom C4'
             next chain B residue 2105 atom C3'
             next chain B residue 2105 atom O3'
             value 82.08 sigma 7.736780088252 period 1
…
```

Phenix restraints snippet:

```
…
dihedral {
    action = *ntc_delete
    atom_selection_1 = $B_G_2105 and name C5'
    atom_selection_2 = $B_G_2105 and name C4'
    atom_selection_3 = $B_G_2105 and name C3'
    atom_selection_4 = $B_G_2105 and name O3'
  }
dihedral {
    action = *ntc_change
    atom_selection_1 = $B_G_2105 and name C5'
    atom_selection_2 = $B_G_2105 and name C4'
    atom_selection_3 = $B_G_2105 and name C3'
    atom_selection_4 = $B_G_2105 and name O3'
    angle_ideal = 82.08
    sigma = 15.47
    periodicity = -1
}

…
```

Note: These DNATCO-generated restraints require a customized version of PHENIX.

Buster restraints snippet:

```
NOTE BUSTER_UTILTOR 1 82.1 7.737 B|2105:C5' B|2105:C4' B|2105:C3' B|2105:O3'
NOTE BUSTER_UTILTOR 1 206.3 11.757 B|2105:C4' B|2105:C3' B|2105:O3' B|2106:P
NOTE BUSTER_UTILTOR 1 287.9 9.803 B|2105:C3' B|2105:O3' B|2106:P B|2106:O5'
NOTE BUSTER_UTILTOR 1 293.5 9.345 B|2105:O3' B|2106:P B|2106:O5' B|2106:C5'
NOTE BUSTER_UTILTOR 1 172.6 10.129 B|2106:P B|2106:O5' B|2106:C5' B|2106:C4'
NOTE BUSTER_UTILTOR 1 54.9 9.598 B|2106:O5' B|2106:C5' B|2106:C4' B|2106:C3'
NOTE BUSTER_UTILTOR 1 81.9 6.956 B|2106:C5' B|2106:C4' B|2106:C3' B|2106:O3'
NOTE BUSTER_UTILTOR 1 198.7 10.907 B|2105:O4' B|2105:C1' B|2105:N9 B|2105:C4
NOTE BUSTER_UTILTOR 1 200.4 10.330 B|2106:O4' B|2106:C1' B|2106:N9 B|2106:C4
NOTE BUSTER_DISTANCE 5.450 0.341 B|2105:C4 B|2106:C4
NOTE BUSTER_DISTANCE 4.767 0.375 B|2105:N9 B|2106:N9
```

References

1. Berman, H.M., Battistuz, T., Bhat, T.N., Bluhm, W.F., Bourne, P.E., Burkhardt, K., Feng, Z., Gilliland, G.L., Iype, L., Jain, S. *et al.* (2002) The Protein Data Bank. *Acta Crystallogr D Biol Crystallogr*, **58**, 899-907.

2. Berman, H., Henrick, K. and Nakamura, H. (2003) Announcing the worldwide Protein Data Bank. *Nat Struct Biol*, **10**, 980.

3. Joosten, R.P., Long, F., Murshudov, G.N. and Perrakis, A. (2014) The PDB_REDO server for macromolecular structure model optimization. *IUCr J*, **1**, 213-220.

4. de Vries, I., Kwakman, T., Lu, X.J., Hekkelman, M.L., Deshpande, M., Velankar, S., Perrakis, A. and Joosten, R.P. (2021) New restraints and validation approaches for nucleic acid structures in PDB-REDO. *Acta Crystallogr D Struct Biol*, **77**, 1127-1141.

5. Tishchenko, S., Kostareva, O., Gabdulkhakov, A., Mikhaylina, A., Nikonova, E., Nevskaya, N., Sarskikh, A., Piendl, W., Garber, M. and Nikonov, S. (2015) Protein-RNA affinity of ribosomal protein L1 mutants does not correlate with the number of intermolecular interactions. *Acta Crystallogr D Biol Crystallogr*, **71**, 376-386.

6. Sarver, M., Zirbel, C.L., Stombaugh, J., Mokdad, A. and Leontis, N.B. (2008) FR3D: finding local and composite recurrent structural motifs in RNA 3D structures. *J Math Biol*, **56**, 215-252.

7. Jiří Černý, Robert A. Nicholls, Dariusz Brzezinski, Helen M. Berman, Miroslaw Gilski, Robbie P. Joosten, Marcin Kowiel, Catherine L. Lawson, Nigel W. Moriarty, Jane S. Richardson *et al.* (2025) NEW TARGETS AND PROCEDURES FOR VALIDATING THE VALENCE GEOMETRY OF NUCLEIC ACID STRUCTURES. *To Be Submitted to Nucleic Acids Research*.

8. Murshudov, G., Dodson, E. and Vagin, A. (1996) In Dodson, E., Moore, M., Ralph, A. and Bailey, S. (eds.), *Proceedings of the CCP4 study weekend: macromolecular refinement*. CCLRC Daresbury Laboratory, Warrington, UK, pp. 93-104.

9. Agirre, J., Atanasova, M., Bagdonas, H., Ballard, C.B., Basle, A., Beilsten-Edmands, J., Borges, R.J., Brown, D.G., Burgos-Marmol, J.J., Berrisford, J.M. *et al.* (2023) The CCP4 suite: integrative software for macromolecular crystallography. *Acta Crystallogr D Struct Biol*, **79**, 449-461.

10. Roversi, P., Blanc, E., Vonrhein, C., Evans, G. and Bricogne, G. (2000) Modelling prior distributions of atoms for macromolecular refinement and completion. *Acta Crystallogr D Biol Crystallogr*, **56**, 1316-1323.

11. Liebschner, D., Afonine, P.V., Baker, M.L., Bunkoczi, G., Chen, V.B., Croll, T.I., Hintze, B., Hung, L.W., Jain, S., McCoy, A.J. *et al.* (2019) Macromolecular structure determination using X-rays, neutrons and electrons: recent developments in Phenix. *Acta Crystallogr D Struct Biol*, **75**, 861-877.

12. Tishchenko, S., Kostareva, O., Gabdulkhakov, A., Mikhaylina, A., Nikonova, E., Nevskaya, N., Sarskikh, A., Piendl, W., Garber, M. and Nikonov, S. (2015) Protein-RNA affinity of ribosomal protein L1 mutants does not correlate with the number of intermolecular interactions. *Acta Crystallogr D Biol Crystallogr*, **71**, 376-386.
